# Supplementary figures and images for: Geolocator tagging links distributions in the non-breeding season to population genetic structure in a sentinel North Pacific seabird
Source: PLoS One. 2020 Nov 9;15(11):e0240056. doi: 10.1371/journal.pone.0240056 (PMC7652296; doi:10.1371/journal.pone.0240056)

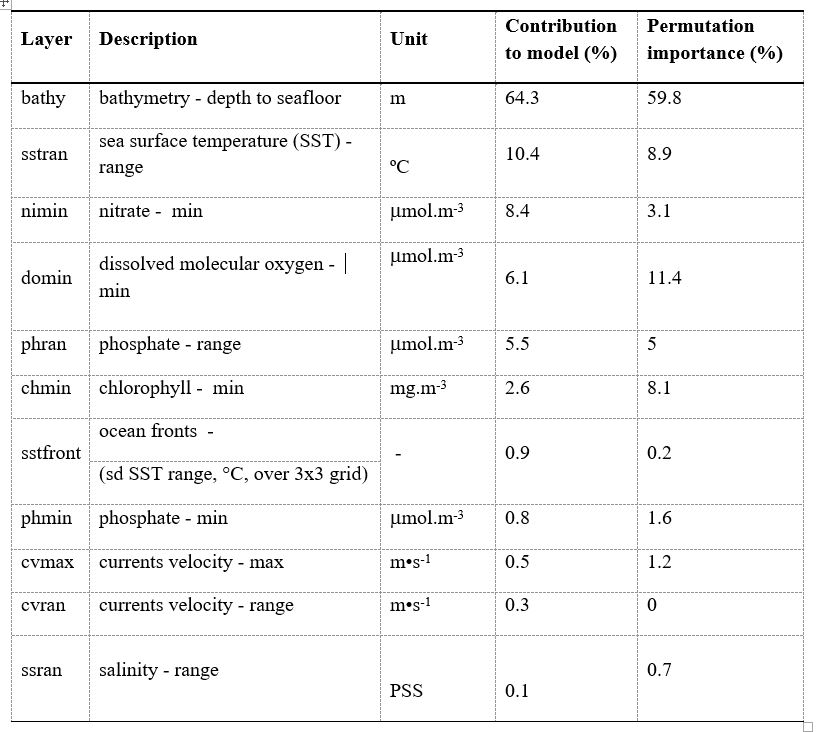

Supplement: S1 Table — Contributions to the model are determined using a heuristic approach that depends on the path of the Maxent code. Permutation importance is determined by values randomly permutated along training points and measurements for the decrease in training AUC. Variables with a higher influence have a larger percent value. (DOCX) [file pone.0240056.s001.docx]

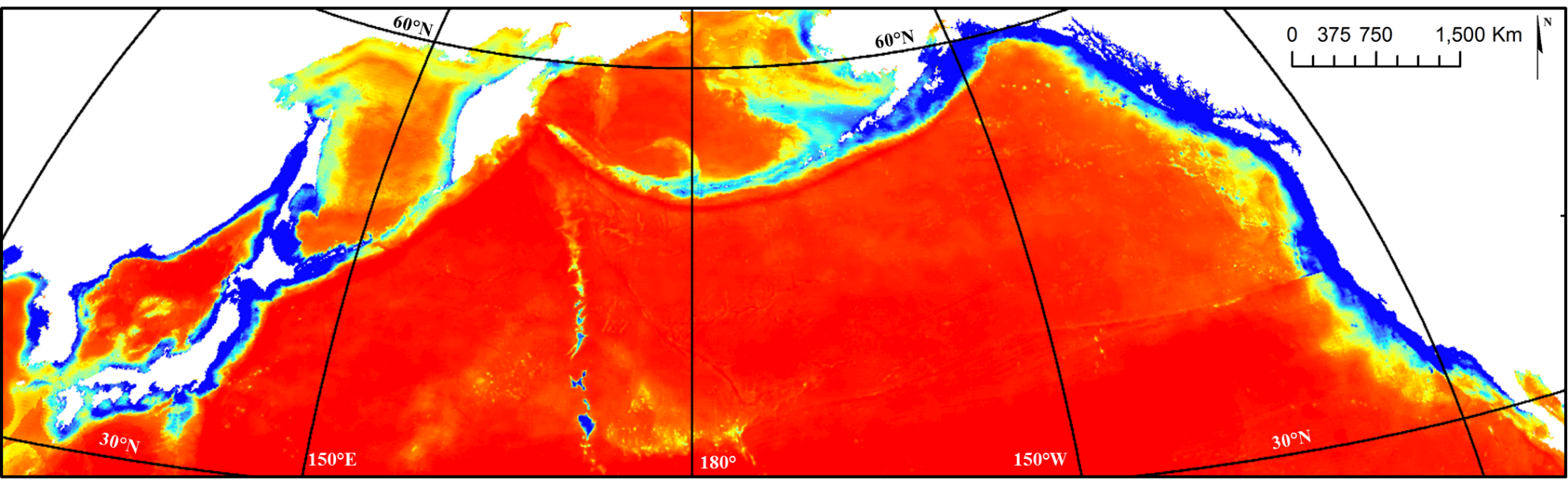

Supplement: S1 Fig — Map was produced using the SDM toolbox (Brown, 2014; Brown et al., 2017), Maxent (Phillips et al., 2006; Phillips & Dudík, 2008), and ArcMap. The final map was visualised using ArcMap 10.2 (ESRI®) in the Azimuthal Equidistant (180° meridian) projection. Most suitable habitat for is shown in cool (blue) unsuitable habitat in warm (orange to red). (TIF) [file pone.0240056.s002.tif]

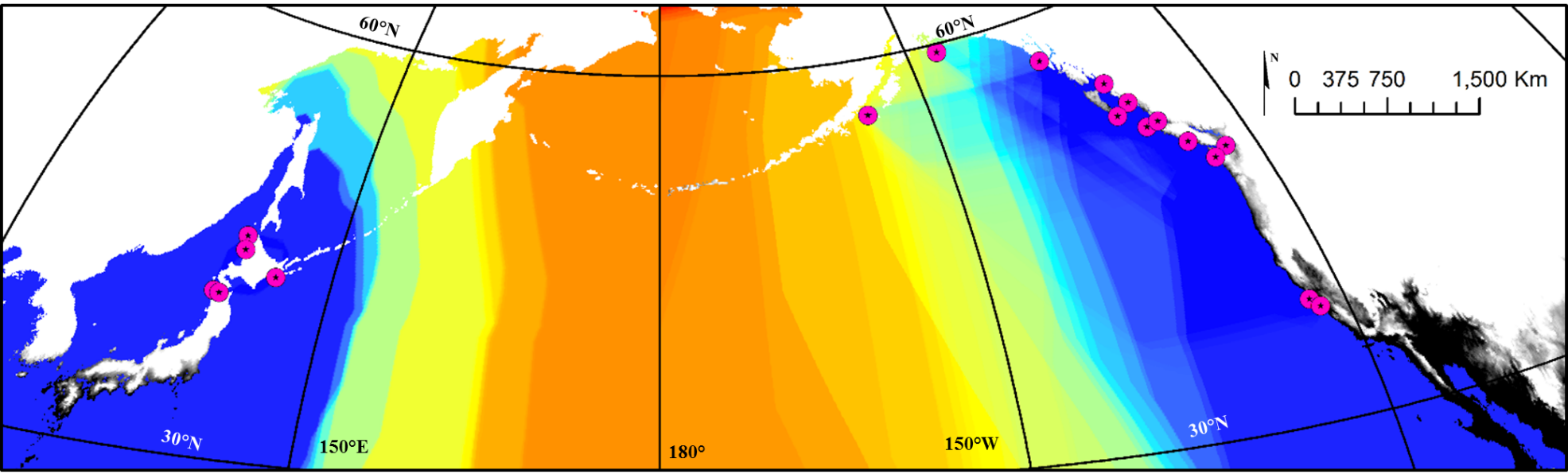

Supplement: S2 Fig — Areas of low resistance are in blue with gradients of orange to red indicating area of higher resistance. Breeding colonies denoted by the pink circled star. Resistance surfaces were developed using friction surfaces from an inverted SDM for rhinoceros auklet and least-cost corridors function within the SDM toolbox (Brown, 2014; Brown et al., 2017). The final map was visualised using ArcMap 10.2 (ESRI®) in the Azimuthal Equidistant (180° meridian) projection. (TIF) [file pone.0240056.s003.tif]
